# “We’re certainly not in our comfort zone”: a qualitative study of GPs’ dementia-care educational needs

**DOI:** 10.1186/s12875-017-0639-8

**Published:** 2017-05-22

**Authors:** Tony Foley, Siobhán Boyle, Aisling Jennings, W. Henry Smithson

**Affiliations:** Department of General Practice, Western Gateway Building, University College Cork, Cork, Ireland

**Keywords:** Dementia, General practitioner, Needs assessment, Patients, Caregivers

## Abstract

**Background:**

Rising dementia prevalence rates rise combined with the policy objective of enabling people with dementia to remain living at home, means that there will be a growing demand for dementia care in the community setting. However, GPs are challenged by dementia care and have identified it as an area in which further training is needed. Previous studies of GPs dementia care educational needs have explored the views of GPs alone, without taking the perspectives of people with dementia and family carers into account. The aim of the study was to explore GPs’ dementia care educational needs, as viewed from multiple perspectives, in order to inform the design and delivery of an educational programme for GPs.

**Methods:**

A qualitative study of GPs, people with dementia and family carers in a community setting was undertaken. Face-to-face interviews were performed with GPs, people with dementia and with family carers. Interviews were audio-recorded, transcribed verbatim and thematically analysed.

**Results:**

Thirty-one people were interviewed, consisting of fourteen GPs, twelve family carers and five people with dementia. GPs expressed a wish for further education, preferentially through small group workshops. Five distinct educational needs emerged from the interviews, namely, diagnosis, disclosure, signposting of local services, counselling and the management of behavioural and psychological symptoms (BPSD). While GPs focused on diagnosis, disclosure and BPSD in particular, people with dementia and family carers emphasised the need for GPs to engage in counselling and signposting of local services.

**Conclusions:**

The triangulation of data from multiple relevant sources revealed a broader range of GPs’ educational needs, incorporating both medical and social aspects of dementia care. The findings of this study will inform the content and delivery of a dementia educational programme for GPs that is practice-relevant, by ensuring that the curriculum meets the needs of GPs, patients and their families.

## Background

The central role of general practitioners (GPs) in the care of patients with dementia has been highlighted in the Irish National Dementia Strategy [1], echoing national dementia strategies across Europe [2, 3] and has also been emphasized in major dementia care guidelines [4, 5]. Rising dementia prevalence rates combined with the policy objective of enabling people with dementia to remain living at home, means that there will be a growing demand for community-based dementia care in the future. In common with other medical professionals, nurses and allied healthcare professionals, GPs are challenged by the complexities of dementia care [6]. Low rates of diagnosis, inappropriate specialist referral and sub-optimal management of dementia in general practice, have been reported [7, 8]. GPs have described barriers to care that include inadequate support services, a lack of time, financial constraints, concerns regarding stigma and also diagnostic and disclosure uncertainty [9].

In order to address these barriers to care, further education of GPs has been widely advocated [10, 11]. GPs themselves have also identified dementia care as an area of significant educational need [12–14]. However, educational interventions have had mixed results in terms of improving GPs’ dementia care knowledge and practice, highlighting the need for a better understanding of how to support GPs in their delivery of optimal care.

For instance, a multifaceted intervention employing interactive seminars, a website and also collaboration with a case manager found that the effects of the educational sessions were weak, reporting that there were few differences in GPs’ knowledge or attitude favorable to dementia care [15]. Similarly disappointing findings were reported in a French study, in which GPs participated in a 2-hour meeting on dementia and received training from specialists in the use of a battery of four neuropsychological tests [16]. In contrast, somewhat more promising results were found in a study undertaken in the UK in which GPs were randomly assigned to one of three intervention arms, a small group workshop, decision support software or an electronic tutorial on CD-ROM [17]. The detection of dementia increased in the decision support software arm and in the practice-based workshops arms although there was no improvement in GPs’ adherence to dementia care guidelines. The authors concede that study power was reduced due to a relatively low number of cases of dementia identified after the study intervention and the relatively few cases in the control arm. Systematic reviews of educational interventions in dementia care have concluded that, in order to promote a positive change in GPs’ practice, educational interventions need to incorporate interactive, facilitated small-group work [18, 19].

These reviews have also highlighted the importance of designing interventions that are tailored to the individual needs of practitioners, a concept which stems from adult learning theory [20]. As adult learners, GPs prefer to engage in educational activities that meet their needs. Whenever possible, information on educational needs should be triangulated, i.e. collected from multiple sources, including learners, patients and the wider society in order to distinguish between learners’ perceived needs alone and their true needs [21]. Taking other perspectives into account is particularly apposite in dementia care, where the illness impacts heavily on both the person with dementia and also on their family carers, and where each has a unique viewpoint into the role of the GP [22].

Despite the body of evidence on the importance of a comprehensive triangulated needs assessment, there is a paucity of published literature on the educational needs of GPs that incorporates the views of people with dementia or their family members. The small number of published studies has been largely limited to the perspective of GPs’ alone. Data has been gathered using a variety of approaches to GPs including questionnaires [12, 13, 23] a Delphi consensus group [24], telephone inquiry [25] and a mixed-method approach using questionnaires and interviews with GPs [26]. While undoubtedly valuable in identifying GPs’ perceived educational needs, these approaches do not take the perspectives of those who live with dementia into account, i.e. people with dementia and their family carers.

The aim of this study was to explore GPs’ dementia care educational needs, by analysing information gathered from a variety of relevant sources, in order to inform the development of a primary care dementia educational programme.

## Methods

A qualitative study, involving face-to-face interviews was performed. A qualitative approach is an appropriate methodology when exploring the behaviour, views and experiences of individuals [27]. Face-to-face interviews were chosen, as opposed to focus groups, as the data collection method, in order to achieve in-depth sensitive discussions of dementia care, which can be a complex, emotive area. Data was gathered through interviews with GPs, people with dementia and family carers. The role of families in supporting people with dementia is widely acknowledged and there is a recognition that family carers need to be supported in order to prevent negative physical and mental health consequences of caring [22]. Incorporating the views of these key stakeholders ensured that the identification of GPs’ educational needs was not restricted solely to the perceived educational needs of GPs themselves.

The study was conducted in counties throughout Munster and Leinster, Ireland. Interviews were held in a location suitable to each participant e.g. their GP surgery or their home.

### Ethical considerations

Prior to initiation of the study ethical approval was sought from and granted by the Clinical Research Ethics Committee of the Cork Teaching Hospitals.

### Sampling and recruitment

GPs were considered eligible to participate if they were in current practice and cared for patients with dementia. GPs were identified though the Irish Medical Directory. In order to ensure there was a spread of experience amongst participating GPs, from this list we purposively invited GPs who had qualified in medicine across a broad range of years. We also attempted to ensure a representative variety of practices by identifying and inviting GPs who practiced from a mixture of rural and urban settings. In total eighteen GPs were approached, of whom fourteen agreed to participate.

To support the recruitment of family carers, participating GPs were requested to identify family carers of people with dementia and to ask them to consider being interviewed. Those who expressed an interest were sent invitation letters. Family carers were considered eligible to participate if they either currently or previously had been the primary carer of a person with dementia. Fifteen family carers were approached, of whom twelve agreed to participate.

The Alzheimer Society of Ireland (ASI) facilitated the recruitment of people with dementia. Individuals who had previously expressed a wish to be involved in dementia research were contacted by the ASI and were informed about the study. Those who consented to being contacted were then each phoned by the research team and further briefed on the study, using communication guidance for interviewing people with dementia for the purposes of research [28]. Through this detailed discussion, researchers ensured that potential participants had a full understanding of the process and confirmed their capacity to participate in interviews. People with dementia were considered eligible to participate once they had received a diagnosis of dementia, were aware of this diagnosis and were deemed to have the capacity to participate in interviews with informed consent. Eight people with dementia were approached of whom five agreed to take part.

Written informed consent was gained from all participants across the three groups.

### Data collection

Semi-structured in-depth interviews were conducted using two topic guides, one of which was used for GP interviews and the other for interviews with people with dementia and family carers. These were informed by a review of the literature on how to assess educational needs and were reviewed by medical educationalists and revised.

The topic guides were piloted with two GPs and with one family carer, following which final revisions were made. The main topics for GP interviews are outlined in Table 1, while the main topics for interviews with people with dementia and family carers is outlined in Table 2. Topic guides were modified after each interview in order to pursue emergent themes.

**Table 1:** Main interview topics for interviews with GPs

1.	Identifying core competencies - What a GP needs to know and do to deliver good dementia care
2.	Identifying challenges and solutions - The barriers and facilitators of dementia care
3.	Perceived educational need - What GPs feel they need to learn more about
4.	Delivering the education - The most appropriate strategies for receiving education and training

**Table 2:** Main interview topics for interviews with people with dementia and with family carers

1.	Reflection on experience – How the GP was involved at diagnosis and as dementia progressed.
2.	Identifying core competencies – The aspects of GP care which were helpful
3.	Identifying challenges and solutions – The aspects of GP care which did not work well and how this might be overcome
4.	Perceived educational need – The aspects of dementia care on which GPs should receive further training

The primary author (TF), a GP, interviewed all of the GPs and two family carers while co-author (SB), a researcher with a background in Public Health, interviewed all other family carers and all of the people with dementia. All interviews were audio-recorded and transcribed verbatim. The average interview time was thirty-two minutes.

Interviews with each participant group proceeded until researchers reached a consensus on conceptual thematic data saturation. Thematic saturation appeared to have been reached after eleven interviews with GPs. A further three interviews were performed to confirm data saturation, in accordance with an accepted method of detecting data saturation [29]. No new themes emerged. In interviews with family carers, thematic saturation was reached after nine interviews. Similarly, three further interviews were performed to confirm data saturation. Again, no further themes emerged in these interviews. Five interviews with people with dementia were performed. Saturation was achieved after these five interviews, though no further confirmatory interviews were performed, due to recruitment challenges.

Interviews took place between October 2015 and June 2016. Transcripts of interviews were returned to all interviewees for comment and minor corrections were made.

### Data analysis

Data was analysed iteratively, following the phases of thematic analysis [30]. The initial step involved two independent researchers becoming familiar with the data, reading the first three transcripts, noting ideas and generating initial codes. The researchers met and agreed on these initial codes. Codes were then analysed and collated into potential themes and further reviewed, thereby generating a thematic map of the analysis. Ongoing analysis ensured the refinement of themes and the clarity of the definitions and names of each theme. All remaining interviews were independently analysed and coded by both researchers adhering to the principles of constant comparison [31]. The researchers met on four further occasions to agree refinements of major themes and the development of sub-themes. To enhance intra-coder reliability and verify emergent themes and sub-themes, three transcripts were randomly chosen and analysed independently by a third researcher (AJ), a GP. Minor disagreement arose in the categorization of some sub-themes, which was remedied through further team discussion.

N Vivo 10 qualitative data software was used for data analysis and management. The authors adhered to the consolidated criteria for reporting qualitative research (COREQ) statement in reporting the findings of the study [32].

## Results

### Characteristics of interviewees

Thirty-one people were interviewed in total, consisting of fourteen GPs, twelve family carers (FCs) and five people with dementia (PwD). The characteristics of each group of participants are shown in Tables 3, 4 and 5.

**Table 3:** Characteristics of GPs (*n* = 14)

Gender	Participants, % (*n*)
Male	64% (9)
Female	36% (5)
Time Qualified	
< 10 years	14% (2)
11–20 years	43% (6)
> 20 years	43% (6)
Practice Location	
Rural	14% (2)
Urban	21% (3)
Mixed	64% (9)
Type of Practice	
Small (1–2 GPs)	36% (5)
Large (3 or more GPs)	64% (9)

**Table 4:** Characteristics of family carers (*n* = 12)

Gender	Participants, % (*n*)
Female	75% (9)
Male	25% (3)
Relationship with PwD	
Wife	25% (3)
Husband	17% (2)
Daughter	50% (6)
Son	8% (1)
Living/Lived with PwD	
Yes	75% (9)
No	25% (3)

**Table 5:** Characteristics of people with dementia (*n* = 5)

Gender	Participants, % (*n*)
Female	60% (3)
Male	40% (2)
Age Range	
< 65 years	60% (3)
> 65 years	40% (2)

### Complexity of dementia care

Generally, GPs regarded dementia as a complex and challenging area of care. One GP contrasted their relative comfort in managing other chronic diseases compared to their unease in managing dementia.

*‘They come in and we check their blood pressure, we tell them that blood pressure’s up…and we check their pulse and we diagnose atrial fibrillation and we’re doing CHAD-Vascing, and we’re in our comfort zone. But we’re certainly not in our comfort zone with this’* (GP5)

While many family carers and people with dementia acknowledged the central role played by GPs in dementia care,

*…because GPs are obviously the front line in most situations* (PwD 3)

some of them also expressed concerns about their GPs’ lack of dementia knowledge.

‘*In many ways in was heart-breaking the way it happened in that the GP who was concerned in the case at the time, wasn’t sufficiently well up on the condition of dementia’* (FC1)

### The educational needs of GPs: main themes

Regarding the dementia-specific educational needs of GPs, five distinct themes emerged from the interviews;

i. Diagnosis
ii. Disclosure
iii. Signposting of local services and supports
iv. Counselling
v. The management of behavioural and psychological symptoms (BPSD)

1. Diagnosis
  A recurring theme that emerged in interviews with GPs was their difficulty in making a diagnosis of dementia. One GP described the challenge of differentiating between mild cognitive impairment and dementia, explaining the challenge raised by the evolving nature of the diagnosis.
*‘Ok, so initially you’re making your assessment and you’re thinking “Right, is this mild cognitive impairment, is this dementia? It can be a protracted thing before you make your diagnosis’* (GP3)
  Some GPs explained the complexity of interpreting the results of cognitive screening tools.
*‘So this might be somebody who is well educated, they score well on their mini mental state exam (sic), but I’m thinking, well look is that just because they have a high level of education? Or is it that they’re cognitively OK?’* (GP1)
  GPs also described struggling with the decision around if and when to refer for confirmation of the diagnosis of dementia.
*‘… even when patients come in where I’m aware of mild cognitive impairment you do an MMSE and it’s fine, it’s 27 or 28, at what stage to do…do you refer everybody? And should they all be seen at a memory clinic and have their scans done?’* (GP2)
  and
*‘So somebody might come to me and say, “I’m a bit forgetful” and to be honest my heart sinks a bit, because I don’t feel comfortable, I don’t feel like I’ve got a good strategy. I suppose it’s being sure that they have dementia, and when to refer. That would be a big issue.’ (GP5)*
2. Disclosure
  The disclosure of the diagnosis of dementia by the GP was emphasized by a majority of both people with dementia and by family carers as an important step in optimal dementia care, describing the need for GPs to receive training on how to sensitively disclose the diagnosis.
*‘Certainly, I’ve met a lot of people who have had the diagnosis and they went into a kind of shock… and I suppose what I want to say is that GPs could be usefully assisted in dealing with that situation’* (PwD3)
  Many GPs described great difficulty in deciding when and even whether to disclose a diagnosis of dementia, balancing the potential benefit and harm to their patient. One GP explained that sometimes non-disclosure was a better decision, provided that the patient’s care was not compromised.
*‘I think it’s hard to know the initial thing of how much to inform the patient. Sometimes you do get the impression that the patient is better off not having a formal diagnosis… But as long as the patient is benefiting from that, that their care is going to be maximized’* (GP8)
3. Signposting of local services and supports
  The need for GPs to provide information was widely recounted by all groups of interviewees, in particular guidance towards local community-based health and social care services and supports.
**Healthcare services**
  Many family carers described the value of the role played by primary care team members and the need for GPs to direct them towards these healthcare professionals.
  ‘*Oh, yeah, information is a big thing that GPs could provide for carers of people with dementia. Like show them the right path for the district nurse, the occupational therapist, you know? It would be a big help for them. It’s all new to them as well.’* (FC5)
  Despite recognizing the importance of a multidisciplinary approach, many GPs were unsure of where these services were or of how to access services.
*I would probably struggle a bit in identifying the wider team’* (GP1)
  Family carers and GPs in particular emphasized the need for respite, home-help and day-care services in the community.
*The other thing we had for my Mum was a day care centre and that was invaluable; it was absolutely fantastic. She loved going there’* (FC3)
  and
*‘I think you live on this lifeline of getting this respite and that helps you to cope as a carer’* (GP5)
  However some family carers felt that they had not been directed to services that were available by their GP.
*‘I suppose what kind of facilities might be available, like home care – something I was oblivious to really. I never went to the trouble of finding out I suppose and so like it was trial and error really’* (FC2)
**Social care services**
  Family carers and people with dementia, in particular, emphasized the value of social supports and felt that their GP should signpost these services to them
*‘…getting onto the ASI helpline, these are the booklets, these are helpful, simple things that you can read, as you are able in helping your loved one and equally so that should be for the GP – they should be signposting’* (PwD1)
  One family carer displayed frustration at the lack of information being imparted,
*‘…and they’re completely unaware of what is out there, and their GP is not telling them’* (FC4).
  Again, many of the GPs struggled to know how to access these services locally.
*‘I suppose, I know that the Alzheimer’s Association, that there is some support available through that, but really being honest about it, I know that we have a very vague idea of that, and I would direct them towards that group, but I myself wouldn’t be able to provide the specifics of it’* (GP1)
**Legal & Financial**
  GPs recognized their role in referring for legal and financial advice.
*‘From a legal perspective it’s important early on to encourage them to look into getting an enduring power of attorney, sorting out financial affairs, discussing it openly with their trusted advisors so that plans may be made for when they’re in a position when they may not be able to manage their affairs’* (GP6)
  Similarly family carers felt GPs had a role in both referring for legal and financial advice and also in giving out information and highlighted the need for proactive management early in the course of the dementia.
*‘But even if they (GPs) are not prepared to discuss it with the family then to have some literature or information to say you need to do this now, while the person is in the full of their health and it needs to be when somebody is in the beginning stages, because otherwise the person, the solicitor isn’t going to be able to do it’* (FC3)
  and
*‘I know that it was just not done in time for my dad; he could barely sign his name at that point’* (FC4)
4. Counselling
**Counselling people with dementia**
  Some GPs acknowledged that dementia caused significant stress for people with dementia especially post diagnosis and felt that they needed to offer support around this time.
*‘I think a big part of my role is actually, maybe, emotional support in terms of helping them deal with what can be a devastating diagnosis’* (GP13)
  While GPs broadly identified their role in supporting patients and family carers, they did not identify counselling as an area of educational need. However, people with dementia and family carers were more explicit in prioritizing the need for GPs to be up-skilled as counselors.
*‘I think that because of the nature of the disease, to my mind, is very much more about the GP being a counsellor in lots of ways’* (PwD3)
**Counselling family carers**
  The impact on a family carer, and the central role of the GP in supporting them, was described by all groups of interviewees.
*‘I think that the carer would be able to manage longer at home with the support of the GP’* (FC1)
  All GPs appeared to be cognisant of this role.
*‘One of the big learnings I’ve had is the carer support and how important carer support is in the management of the patient’* (GP6)
  However, some family carers and people with dementia described feeling isolated and highlighted the issues of GPs’ limited accessibility and time and emphasized the need for GPs to pro-actively initiate and maintain follow-up care.
*‘…maybe a recommendation about the GP initiating calls, maybe initiating an appointment, you know not waiting, because people can sink and lose heart and sink and sink’* (PwD3)
5. The management of behavioural and psychological symptoms (BPSD)
  Many GP interviewees described being challenged by the management of BPSD in their patients with advancing dementia.
*‘Some of the behavioural symptoms can be very difficult to deal with…aggression, wandering, incontinence, sexual disinhibition’* (GP7)
  In particular, GPs perceived their role as being a prescriber of medications to manage BPSD, describing prescribing dilemmas,
*‘As things progress from moderate to more severe, the behavioural management with medication, I think we all just kind of make a stab here and there at what we feel might be appropriate, do you know?’* (GP11)
  GPs expressed a need for more education around the prescribing of psychotropic medication.
*‘When do you add in psychotropic medication, what type of medication, what dosages, for how long? We need guidelines on that’* (GP3)
  Some family carers also felt that GPs should have a role in guiding them towards strategies for the non-pharmacological management of BPSD.
*‘…I had to develop a technique to try to snap him out of that, and I used to try reminiscing and that kind of thing and that worked, but I learnt all that myself through the internet, to be quite honest with you ….in every surgery I think that there should be a list of what could be available’* (FC1)
**Further education**
  All GPs expressed a wish for further dementia education and training. Regarding mode of delivery, the majority of GPs had a preference for small group workshops.
*‘I do enjoy a group setting, you know, a discussing setting rather than a didactic session’* (GP13)
  The other mode of delivery favoured by some GPs was on-line learning, having readily accessible desktop guidelines for dementia care.
*‘Guidelines would be great, and probably guidelines that are on your computer rather than ones that are up on the shelf someplace. So that would be great’* (GP3)

## Discussion

### Summary of main findings

This qualitative study demonstrated a range of areas within dementia-care in which GPs have an identified educational need. It highlighted GPs’ uncertainties around key aspects of dementia care and explored the complexity of their decision-making. In particular, GPs want more training on diagnosis, disclosure and the management of BPSD. They also indicated that dementia care is an area of significant educational need and showed a strong willingness to engage in further training.

The triangulation of data from family carers and people with dementia revealed a stronger emphasis on the need for GPs to learn about counselling and sign-posting of local services and supports. Both family carers and people with dementia reiterated the value of sensitive disclosure and emphasized the need for proactive GP care, better access to their GP and more time in the consultation. This highlights the need for policy makers to recognize dementia care as a chronic disease that needs to be resourced adequately in primary care.

### Strengths and limitations

The study used a qualitative design, encouraging interviewees to express their own views on the complex topic of dementia care. All interviews were analysed systematically and independently by two analysts and consensus achieved over multiple assessments, ensuring greater precision of definitions and clarity of themes. The triangulation of data from multiple sources enhances the validity of our findings. This approach has been widely adopted and developed as a means of investigating the convergence of the data and also the conclusions derived from the data [33]. This ensured that identified needs were not restricted to the perceived educational needs of GPs alone. We do acknowledge that in some instances patients and family carers may be describing their own unmet needs, as opposed to the true educational needs of GPs. However, this triangulation of perspectives does strengthen the credibility of our findings. Additionally, when analysing optimal care in service delivery, it is important to capture the views of service users [34] and the valuable contributions of people with dementia and family carers thus contributed to the validity of our study.

The external validation of our findings was enhanced by seeking feedback from interviewees on the transcripts of their interviews and by then making minor alterations in response to this feedback.

Regarding the limitations of our study, it is possible that the interviewee responses were influenced by the interviewers, the first of whom is a GP, the second a researcher who has a background in Public Health. The first interviewer interviewed all of the GPs. GPs interviewed by a fellow GP may have felt obliged to give socially desirable answers, thus introducing interviewer bias. However, GP researchers have been shown to gain richer data than non-clinicians when interviewing fellow GPs, as interviewees open-up, permitting themselves a degree of vulnerability [35]. We have endeavoured to take this effect into consideration by taking a teamwork approach when analyzing and interpreting the data [36]. The second researcher interviewed all of the people with dementia and the majority of the family carers, as it was felt that a GP-interviewer may have overly influenced their discussions about their own GP. Furthermore, all except two of these interviews were performed in interviewees own homes in an effort to avoid the potential bias of an interview being carried out in their own GPs’ surgery.

Convenience sampling was employed in order to recruit both family carers and people with dementia, which may have introduced bias. Family carers nominated by their GPs may have had a better relationship with their GPs, while people with dementia recruited via the ASI may have had a greater expertise in dementia care than their peers. However, this expertise may also have offered a better insight into their lived experience with dementia. Further sampling might have been considered via memory clinics or day-care services, in order to include a random sample of people with dementia and family carers, who may not have as close a relationship with their family doctor.

### Comparison with other studies

To the best of our knowledge, this is the first study examining GPs’ dementia care educational needs in which data has been triangulated and integrated from multiple sources.

In line with our findings, previous questionnaire based studies found that GPs’ lacked confidence in diagnosis, disclosure and the management of BPSD and had limited knowledge of local support services [12, 13, 23, 24]. These findings regarding diagnostic uncertainty are not consistent with the findings of a Dutch study, which concluded that GPs are moderately good at recognizing dementia, comparable to dementia identification at a memory clinic [37]. However, that study also found that subtyping of the dementia was a challenge for GPs and recommended diagnostic support from specialist teams.

Our study also highlights that patients and family members felt that GPs needed to engage more in counselling, although few GPs flagged this as an area of personal educational need. Our finding regarding the need for better access to GPs and increased consultation time is supported by other studies beyond dementia care, which found that older patients and their families value these aspects of care, in particular preferring close contact with their family doctor and a more personal model of care [38, 39]. These needs may reflect their greater levels of social isolation and morbidity.

## Conclusions

The findings from this study has the potential to contribute to the design and delivery of dementia training that addresses the perceived educational needs of GPs and meets the expressed needs of people with dementia and their family carers

While the curriculum for this training should include the more traditional medical aspects of dementia care such as diagnosis, disclosure and BPSD it should also incorporate social aspects of dementia care, which are highly valued by patients and their families. Regarding these social aspects of care, priority needs to be given to developing clear local pathways of dementia care in the community, readily accessible by GPs and by their patients.

Informed by the findings of this educational needs analysis, the PREPARED team (Primary Care, Education, Pathways and Research of Dementia) in the Department of General Practice in University College Cork has designed and commenced peer-facilitated, practice-based workshops for GPs nationally and has launched an educational website, www.dementiapathways.ie [40], that includes a directory of local dementia care services and supports. Recognising the importance of interprofessional dementia education, the PREPARED project has also commenced interdisciplinary workshops with other allied healthcare professionals, including occupational therapists, physiotherapists, community nurses and speech and language therapists. An evaluation of this programme is ongoing and will be reported in due course.

Our triangulated data gathering approach, in which the views of patients and their family members are considered may also be applicable to other fields beyond dementia care, in particular in the management of other chronic diseases in primary care. In order to meet the needs of service users, future research on clinicians’ educational needs should include the expert views of patients and, where appropriate, their families too.

## Abbreviations

ASI: Alzheimer society of Ireland
BPSD: Behavioural and psychological symptoms of dementia
COREQ: Consolidated criteria for reporting qualitative research
FC: Family carer
GP: General practitioner
PREPARED: Primary care, education, pathways and research of dementia
PwD: People with dementia
